# Incorporation and influence of *Leishmania* histone H3 in chromatin

**DOI:** 10.1093/nar/gkz1040

**Published:** 2019-11-13

**Authors:** Mariko Dacher, Hiroaki Tachiwana, Naoki Horikoshi, Tomoya Kujirai, Hiroyuki Taguchi, Hiroshi Kimura, Hitoshi Kurumizaka

**Affiliations:** Laboratory of Chromatin Structure and Function, Institute for Quantitative Biosciences, The University of Tokyo, 1-1-1 Yayoi, Bunkyo-ku, Tokyo 113-0032, Japan; Department of Cancer Biology, The Cancer Institute of Japanese Foundation for Cancer Research, 3-8-31 Ariake, Koto-ku, Tokyo 135-8550, Japan; Graduate School of Advanced Science and Engineering, Waseda University, 2-2 Wakamatsu-cho, Shinjuku-ku, Tokyo 162-8480, Japan; Laboratory of Chromatin Structure and Function, Institute for Quantitative Biosciences, The University of Tokyo, 1-1-1 Yayoi, Bunkyo-ku, Tokyo 113-0032, Japan; Graduate School of Advanced Science and Engineering, Waseda University, 2-2 Wakamatsu-cho, Shinjuku-ku, Tokyo 162-8480, Japan; Cell Biology Center, Institute of Innovative Research, Tokyo Institute of Technology, 4259 Nagatsuta-cho, Midori-ku, Yokohama 226-8503, Japan; Laboratory of Chromatin Structure and Function, Institute for Quantitative Biosciences, The University of Tokyo, 1-1-1 Yayoi, Bunkyo-ku, Tokyo 113-0032, Japan; Graduate School of Advanced Science and Engineering, Waseda University, 2-2 Wakamatsu-cho, Shinjuku-ku, Tokyo 162-8480, Japan

## Abstract

Immunopathologies caused by *Leishmania* cause severe human morbidity and mortality. This protozoan parasite invades and persists inside host cells, resulting in disease development. *Leishmania* modifies the epigenomic status of the host cells, thus probably averting the host cell defense mechanism. To accomplish this, *Leishmania* may change the host cell chromatin structure. However, the mechanism by which the parasite changes the host cell chromatin has not been characterized. In the present study, we found that ectopically produced *Leishmania* histone H3, LmaH3, which mimics the secreted LmaH3 in infected cells, is incorporated into chromatin in human cells. A crystallographic analysis revealed that LmaH3 forms nucleosomes with human histones H2A, H2B and H4. We found that LmaH3 was less stably incorporated into the nucleosome, as compared to human H3.1. Consistently, we observed that LmaH3–H4 association was remarkably weakened. Mutational analyses revealed that the specific LmaH3 Trp35, Gln57 and Met98 residues, which correspond to the H3.1 Tyr41, Arg63 and Phe104 residues, might be responsible for the instability of the LmaH3 nucleosome. Nucleosomes containing LmaH3 resisted the Mg^2+^-mediated compaction of the chromatin fiber. These distinct physical characteristics of LmaH3 support the possibility that histones secreted by parasites during infection may modulate the host chromatin structure.

## INTRODUCTION

Parasites of the genus *Leishmania* are the causative agents of the immunopathologies known as leishmaniasis. Depending on the parasite species, the clinical manifestations of this disease range largely from self-healing ulcerative skin lesions to disseminated visceral infections that are often fatal (for a recent review, see 1). Leishmaniasis is an endemic disease in tropical and subtropical regions, and constitutes a serious health problem (1,2). At present, there are no vaccines available and the current anti-leishmanial treatments are quite limited due to their toxicity and cost, and their continuous use is threatened by the rise of drug-resistant parasites (1,2). A better understanding of the biology of the host-*Leishmania* interaction would facilitate the discovery of novel targets for anti-leishmanial chemotherapy.

Transmission of *Leishmania* to the mammalian host occurs during a blood meal by infected sand flies. *Leishmania* differentiates and proliferates inside macrophages, and has established efficient strategies to alter the host innate immune response, favoring its survival (3–5).

Chromatin has been increasingly recognized as an important target for many pathogens (reviewed in 6,7). Several reports have found that parasite infection results in the epigenetic modulation of host cells (6–8). For example, Leng *et al.* (9) demonstrated that, during infection, the *Toxoplasma* parasite alters the proinflammatory cytokine production that prevents its transcription, by targeting the histone modification machinery (9). The cattle parasite *Theileria* exhibited an alteration of gene expression favorable for pathogenesis, including genes related to chromatin remodeling, during host cell infection (10). These findings highlight the importance of the host chromatin as the target of numerous pathogens.

With the aim to identify the effector proteins that subvert the host immune response, potential virulence factors secreted by *Leishmania* into the host environment have been investigated in depth (reviewed in 11,12). In early studies, various molecules were reported as secreted factors, including the surface abundant protease of *Leishmania* GP63 (13–15), and the elongation factor-1α homologue (16,17). Recent proteomics study identified numerous proteins secreted by *Leishmania* under defined conditions (reviewed in 11). Intriguingly, histones were frequently found among the identified proteins (17–20). Moreover, infections by other parasites, such as *Trypanosoma* and *Plasmodium*, also cause histone secretion (21–24).

Histone proteins, H2A, H2B, H3 and H4, assemble as a core complex called the nucleosome, which is the fundamental unit of the chromatin in eukaryotic cells (25). The nucleosome wraps ∼150 bp of DNA (26). The structure of chromatin plays an important role in DNA packaging, and regulates gene expression. The histones have diverse variants that build nucleosomes with distinct architectures, resulting in different higher order chromatin configurations (27). Histones are highly conserved among species; for example, yeast histones share 80–92% identity with human histones (28,29). On the other hand, *Leishmania* histones are evolutionally diversified, and have 48–60% identity with human histones (30). Therefore, *Leishmania* histones may affect the chromatin structure, when they are incorporated into the host chromosomes.

In the present study, we ectopically produced *Leishmania major* histone H3 (LmaH3) in human cells, and found that LmaH3 is incorporated into human chromatin. We reconstituted a nucleosome containing LmaH3, together with the human histones H2A, H2B and H4, and determined its crystal structure. We found that LmaH3 destabilizes the nucleosome, probably by the weakened H3–DNA interaction in the nucleosome. A sedimentation velocity assay revealed that chromatin containing LmaH3 was resistant against chromatin compaction by Mg^2+^ ion. These results suggest that the parasite-mediated histone hijacking of the host chromatin may be important for the invasion and persistence of the pathogen inside the host cells, by changing the chromatin structure and dynamics.

## MATERIALS AND METHODS

### Multiple sequence alignment

The *Leishmania major* gene and protein sequences were retrieved from the GeneDB web database (https://www.genedb.org) (31). The human and yeast gene and protein sequences were retrieved from the NCBI Protein database (http://www.ncbi.nlm.nih.gov/protein/). Homology searches were performed using the BLAST program, with the default BLOSUM-62 substitution matrix. The multiple sequence alignment of human H3.1 and LmaH3 was performed using the built-in algorithm ClustalXv2. Additional sequence analyses were performed using the programs in the BioEdit program suite (Tom Hall, North Carolina State University).

### Analysis of LmaH3-GFP incorporation into HeLa cell chromatin

The HeLa cells expressing either H3.1-GFP or LmaH3-GFP were generated using the PiggyBac transposon vector system (System Biosciences). The DNA sequence of *L. major* H3 [LmjF.10.0870 (XP_001681421.1)], designated as LmaH3, was inserted into the PB533A-2 vector (System Biosciences) using the restriction enzymes *Xba*I and *Xho*I, at the N-terminus of the green fluorescent protein (GFP) sequence, to generate the plasmid PB533A-2-LmaH3-GFP, in which LmaH3 is fused to GFP. The plasmid PB533A-2-H3.1-GFP, containing the DNA sequence of human histone H3.1 (NP_003520.1) fused to the GFP tag, was previously described (32). The resulting plasmids were co-transfected into HeLa cells with the PiggyBac transposase vector (PB210PA-1), using Lipofectamine 2000 (Thermo Fisher Scientific). To establish stable cells expressing H3.1-GFP or LmaH3-GFP, referred to as the H3.1-GFP and LmaH3-GFP lines, respectively, the transfected cells were selected with 1 mg/ml G418 (Nacalai Tesque). HeLa cells were cultured as described previously (33). A cell sorter, SH800Z (Sony), was used to select GFP-positive cells.

HeLa cells stably expressing H3.1-GFP or LmaH3-GFP and untransfected control HeLa cells were collected and resuspended in 500 μL of NB buffer (15 mM Tris-HCl (pH 7.5), 15 mM NaCl, 60 mM KCl, 300 mM sucrose and 1× complete EDTA-free Protease Inhibitor (Roche Diagnostics)). Subsequently, 500 μl of NB buffer containing 1% Nonidet *P*-40 was added, and the mixture was rotated for 5 min at 4°C. After centrifugation (1500 × *g*; 5 min; 4°C), the supernatant was removed, and the nuclei were resuspended in 100 μl NB. CaCl_2_ was added to a final concentration of 2 mM, and then the solution was treated with micrococcal nuclease (TaKaRa) for 5 min at 37°C. The reaction was stopped with 10 mM EDTA (pH 8.0), and the solubilized chromatin fragments were collected by centrifugation at 20 000 × *g* for 5 min at 4°C. The samples were incubated for 2 h at 55°C, to denature the nonnucleosomal proteins. After the precipitated proteins were removed by centrifugation (20 000 × *g*; 5 min; 4°C), the supernatant was collected as the nucleosome sample. The nucleosome sample (140 μl) was then fractionated on an 11.5 ml sucrose gradient (10–30%) by ultracentrifugation (209 541 × *g*; 21 h; 4°C), using a Beckman SW41Ti rotor. Fractions (0.5 ml each) were collected from the top.

For DNA analysis, fractions 1–19 were mixed with SDS (0.2%), and analyzed by 1.5% agarose gel electrophoresis in 1× TAE (40 mM Tris-acetate and 1 mM EDTA) with ethidium bromide staining. For protein analysis, the fractions containing mononucleosomes were fractionated by 12% SDS-PAGE and blotted onto Hybond-P polyvinylidene difluoride (PVDF) membranes (GE Healthcare), using a semidry blotting system (Bio-Rad). The membrane was washed once in PBS-T (PBS containing 0.1% Tween 20), and blocked overnight with 5% skim milk (Nacalai Tesque) in PBS-T. The monoclonal anti-GFP horseradish peroxidase (HRP)-conjugated antibody (GFP antibody (B-2) sc-9996; Santa Cruz Biotechnology; 1:300) was added to the membrane, and incubated for 1 h at room temperature. After three washes of the membrane for 10 min in PBS-T, the GFP signals were detected using an ECL Western Blotting Detection System (GE Healthcare). The fractions of the LmaH3-GFP and untransfected cell lines used for the western blotting analysis were further concentrated (13-fold), using an Amicon Ultra 30 kDa filter (Millipore).

### Fluorescence recovery after photobleaching (FRAP) analyses

Fluorescence recovery after photobleaching (FRAP) was performed according to the method described previously (33), using an FV-1000 confocal microscope (Olympus). Images of the cells stably expressing H3.1-GFP and LmaH3-GFP were acquired every 1.644 s, from 8.22 s before photobleaching to 154.536 s (∼2.5 min) after bleaching. The fluorescence intensities of the bleached areas were measured using *ImageJ* (1.46r) (34). To obtain the relative intensities of the bleached areas, after background subtraction, the net intensities of the bleached areas were normalized to the net intensities of the unbleached areas in each time frame. The relative intensities in each time frame were then normalized to the intensity before bleaching. The values of every three frames were plotted.

### Purification of recombinant histones

The LmaH3 DNA sequence was cloned into the pET15b vector and expressed in *E. coli* BL21 (DE3) cells. The human H2A, H2B, H3.1, H4, H3.1 mutants (Y41W, R63Q and F104M), and LmaH3 histones were purified according to the methods described previously (35,36).

### Reconstitution and purification of nucleosomes

The purified, lyophilized H3.1, H3.1 Y41W, H3.1 R63Q, H3.1 F104M or LmaH3 was dissolved in a denaturing solution, containing 20 mM Tris–HCl buffer (pH 7.5), 7 M guanidine hydrochloride, 1 mM EDTA and 20 mM 2-mercaptoethanol, in the presence of stoichiometric amounts of human histones H2A, H2B and H4. After rotation for 1.5 h at 4°C, the samples were dialyzed 4 times, in a solution containing 10 mM Tris–HCl buffer (pH 7.5), 2 M NaCl, 1 mM EDTA (pH 8.0) and 5 mM 2-mercaptoethanol. The reconstituted histone octamers were isolated by Superdex 200 gel filtration column chromatography (GE Healthcare), as previously described (36). The H3.1 and LmaH3 nucleosomes were then reconstituted with the histone octamer containing either H3.1 or LmaH3, with the palindromic 146 bp α-satellite DNA (26) or the palindromic 145 bp 601L DNA (37), by the salt dialysis method (35,36). Briefly, the DNA fragment and the histone octamer were mixed and dialyzed against 2 M KCl buffer, and the KCl concentration was gradually decreased to 250 mM with a peristaltic pump. The reconstituted nucleosomes were further purified by nondenaturing 6% polyacrylamide gel electrophoresis, using a Prep Cell apparatus (Bio-Rad). The H3.1–H4–DNA, H3.1 Y41W–H4–DNA, H3.1 R63Q–H4–DNA, H3.1 F104M–H4–DNA and LmaH3–H4–DNA complexes without H2A and H2B dimers were reconstituted with the H3.1–H4, H3.1 Y41W, H3.1 R63Q, H3.1 F104M and LmaH3–H4 tetramers and the palindromic 145 bp 601L DNA, by the salt dialysis method.

### Crystallization and structure determination of the LmaH3 nucleosome

The LmaH3 nucleosome was concentrated to 3.0 mg/ml (DNA concentration), and crystallized by the hanging drop method. The LmaH3 nucleosome (1 μl) was mixed with 1 μl of reservoir solution, containing 100 mM sodium acetate (pH 4.6), 140 mM MnCl_2_, 6% 2-propanol and 6% trimethylamine N-oxide dehydrate. The samples were incubated at 20°C. The crystals thus obtained were cryoprotected with a 30% polyethylene glycol 400 solution, containing 100 mM sodium acetate (pH 4.6), 126 mM MnCl_2_ and 2% trehalose, and were flash cooled in liquid nitrogen. Diffraction data were collected from the crystals of the LmaH3 nucleosome at the beamline station BL-1A in KEK (Tsukuba, Japan). The diffraction data were indexed, integrated, scaled, and truncated to 3.63 Å based on the criteria of CC_1/2_ >0.5, using the *XDS* program package (38–41). The scaled data were processed with the CCP4 suite to add the *R*_free_ flag (42). The structure of the LmaH3 nucleosome was determined by the molecular replacement method, using Phaser-MR in the PHENIX suite (43,44). The search model was the structure of the human nucleosome (Protein Data Bank ID: 5AY8) (45). The structure of the LmaH3 nucleosome was refined with the PHENIX suite, and the structural model was built with the program COOT (46,47). The final structure presented no outliers in the Ramachandran plot, as indicated with the *MolProbity* program (Supplementary Table S1) (48). All structural figures were created with the PyMOL software (Schrödinger; http://pymol.org).

### Thermal stability assay

Thermal stability assays (49) were performed to assess the stabilities of the H3.1 and LmaH3 nucleosomes, and the H3.1–H4–DNA and LmaH3–H4–DNA complexes without H2A and H2B molecules, which were all assembled with the palindromic 145 bp 601L DNA. 2.25 μg of nucleosome were mixed with 20 μl of 20 mM Tris–HCl (pH 7.5) buffer, containing 1 mM dithiothreitol, 1 mM EDTA, 100 mM NaCl and 5× SYPRO Orange. The fluorescence signals were acquired with the StepOnePlus™ Real-Time PCR system (Applied Biosystems), with continuous fluorescent measurement starting at 26°C and ending at 95°C (ramping rate of 1°C/min). Raw fluorescence data were computed to normalized values as: (*F*(*T*) − *F*_26°C_)/(*F*_95°C_ − *F*_26°C_), where *F*(*T*), *F*_26°C_ and *F*_95°C_ indicate the fluorescence at a particular temperature, the fluorescence at 26°C, and the fluorescence at 95°C, respectively.

### Preparation of the H3.1 and LmaH3 nucleosome arrays

The nucleosome arrays were prepared by the salt dialysis method, using the purified histone octamers containing either H3.1 or LmaH3 and the DNA fragment with 12 tandems repeats of the 177 bp Widom601 sequence (50). The nucleosome occupancy of the reconstituted nucleosome arrays was assessed by digestion with the restriction enzyme *Sca*I, which cleaves the linker DNA regions of the nucleosome array. Briefly, the reconstituted nucleosome array (100 ng of DNA) was digested by *Sca*I in a 10 μl reaction solution (10 mM Tris–HCl (pH 7.5), 50 mM NaCl, 0.5 mM MgCl_2_ and 0.1 mg/ml BSA) at 22°C for 12 h, and the amount of the mononucleosomes thus generated was estimated by nondenaturing 5% polyacrylamide gel electrophoresis, in 1× TBE buffer (90 mM Tris base, 90 mM boric acid and 2 mM EDTA), with ethidium bromide staining.

### Analytical ultracentrifugation sedimentation velocity assay

The nucleosome arrays (OD_260_ = 0.6–0.8) were dialyzed against a solution containing 10 mM Tris–HCl (pH 7.5), in the presence or absence of 0.6 mM MgCl_2_. The samples were collected and placed into 12 mm path length cells. The analytical ultracentrifugation assay was conducted with a ProteomeLab XL-I centrifuge (Beckman Coulter), using an 8-hole An-50Ti rotor. The samples were incubated for 2 h at 20°C, and were then centrifuged at 22 000 rpm. The absorbance at 260 nm was monitored. The collected data were analyzed by the enhanced van Holde–Weischet method (51), using UltraScanII 9.9, revision 1927 (http:/www.ultrascan.uthscsa.edu). A partial specific volume of 0.65 ml/g was used to determine the sedimentation coefficient (*S*_20,w_).

## RESULTS

### *Leishmania* parasite H3 is incorporated into HeLa cell chromatin

**Figure 1.**
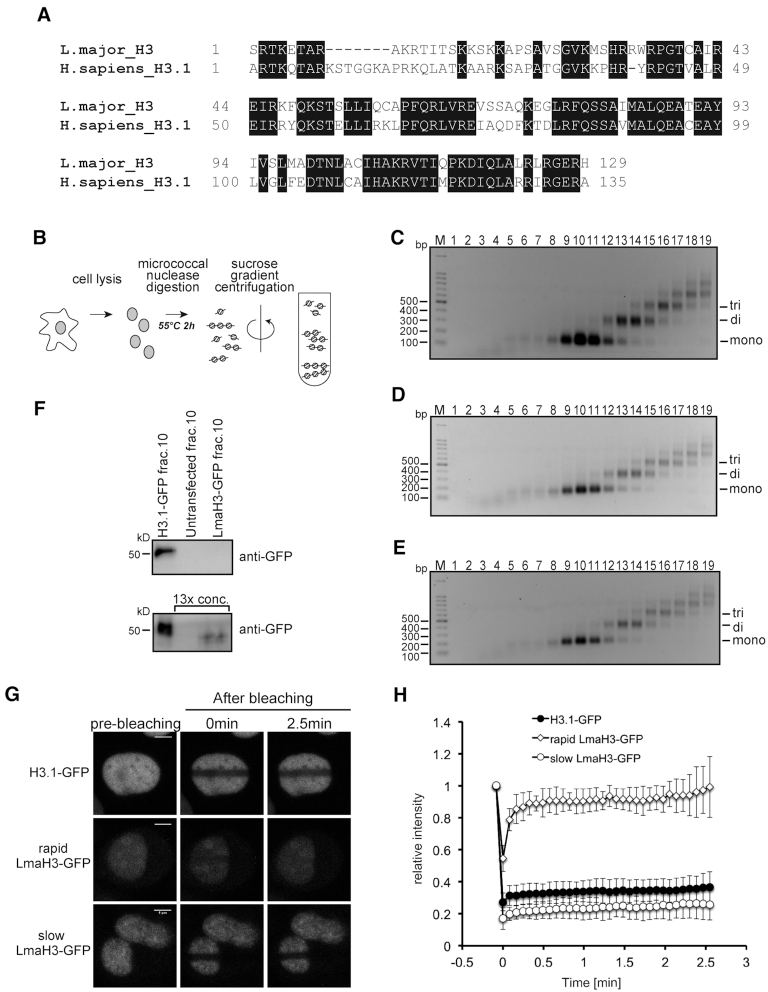
LmaH3 is incorporated into HeLa cell chromatin. (**A**) Amino acid sequence alignment of histone H3s from *Leishmania major* [LmjF.10.0870 (XP_001681421.1)] and *Homo sapiens* (NP_003520.1). The black boxes indicate identical amino acid residues between H3.1 and LmaH3. (**B**) Schematic representation of the fractionation of micrococcal nuclease digested chromatin. (C–E) Sucrose gradient ultracentrifugation. The chromatin fractions from HeLa cells stably expressing LmaH3-GFP (**C**), H3.1-GFP (**D**), or untransfected cells (**E**) were subjected to sucrose gradient ultracentrifugation. The resulting DNA fragments were analyzed by agarose gel electrophoresis with ethidium bromide staining. The DNA markers are indicated as M. (**F**) Detection of LmaH3-GFP by western blotting analysis. The presence of H3.1-GFP and LmaH3-GFP was detected by a western blotting analysis, using the anti-GFP monoclonal antibody. Samples (10 μl each) of HeLa cells expressing H3.1-GFP (fraction 10), untransfected cells (fraction 10), and HeLa cells expressing LmaH3-GFP (fraction 10), were applied (upper panel). To detect the low amount of LmaH3 incorporated into chromatin, samples (fraction 10) from LmaH3-GFP cells and untransfected cells (as a negative control) were concentrated 13-fold, and then subjected to the western blotting. The sample from HeLa cells expressing H3.1-GFP (fraction 10) was not concentrated. The results were reproduced, and are represented in Supplementary Figure S1. The molecular weights of the marker proteins are indicated. The full gel images of Figure 1C–F are presented in Supplementary Figure S5A–E, respectively. (G and H) Fluorescence recovery after photobleaching (FRAP) analysis of HeLa cells expressing H3.1-GFP and LmaH3-GFP. After bleaching a rectangular area of the nucleus, the mobility of H3.1-GFP and LmaH3-GFP in living cells was analysed by monitoring the fluorescence recovery. (**G**) Representative images before photobleaching (left column), upon bleaching at 0 min (centre column), and at 2.5 min (right column) are shown. The images for HeLa cells expressing H3.1-GFP and LmaH3-GFP with fast (rapid LmaH3-GFP) or slow (slow LmaH3-GFP) fluorescence recovery are presented in the upper, middle, and lower panels, respectively. The scale bar indicates 4 μm. (**H**) Graphical representation of the FRAP data. The relative fluorescence intensities of H3.1-GFP (•), rapid LmaH3-GFP (◊), and slow LmaH3-GFP (○) are presented with their standard deviations (*n* = 10).

LmaH3 (XP_001681421.1) consists of 130 amino acids, and shares 60% amino acid identity with human histone H3.1 (H3.1) (Figure 1A). We first tested whether LmaH3 is incorporated into the host chromatin. To do so, we established HeLa cell lines that stably expressed LmaH3 fused to GFP (LmaH3-GFP) and a positive control, H3.1-GFP. Nuclei were isolated from the LmaH3-GFP and H3.1-GFP cell lines, as well as from an untransfected HeLa cell line as a negative control (Figure 1B). Chromatin was recovered in the insoluble fraction, and was treated with micrococcal nuclease. Nucleosomes were recovered in the soluble fraction, and were heated at 55°C for 2 h to denature the non-histone proteins bound to the nucleosomes (Figure 1B). The nucleosomal fractions of the LmaH3-GFP, H3.1-GFP and untransfected HeLa cell lines were separated by sucrose gradient ultracentrifugation (Figure 1C–E, and Supplementary Figure S1). We then assessed whether LmaH3 is present in the mononucleosomal fractions of the host cell chromatin, by immunoblotting using an anti-GFP antibody. Although LmaH3 was scarcely detected in the mononucleosomal fractions without concentration (Figure 1F, upper panel, and Supplementary Figure S1), the LmaH3-GFP signal was clearly detected when the LmaH3-GFP mononucleosomal fraction (fraction 10) was concentrated 13-fold (Figure 1F, lower panel, and Supplementary Figure S1). These results indicated that LmaH3 has the potential to be incorporated into the host cell chromatin.

To test the incorporation of LmaH3-GFP into the host cell chromatin, we compared the mobilities of LmaH3-GFP and H3.1-GFP in living cells, by fluorescence recovery after photobleaching (FRAP). We observed two distinct populations of cells expressing LmaH3-GFP. The first population showed fast fluorescence recovery, indicating the rapid exchange of LmaH3-GFP in chromatin (rapid LmaH3-GFP) (Figure 1G and H). In contrast, the second population suggested the slow exchange of the LmaH3-GFP (slow LmaH3-GFP), similar to that of H3.1-GFP (Figure 1G and H). These results are in good agreement with the data presented in Figure 1F, suggesting that LmaH3 is stably incorporated into the host cell chromatin. Moreover, the slow LmaH3-GFP population was only found in a minor fraction (<1%) of the LmaH3-GFP cells, as compared to the rapid LmaH3-GFP population. This observation may explain the weak intensity of the LmaH3-GFP signal detected by immunoblotting in the mononucleosomal fraction of the host cell chromatin (Figure 1F, upper panel, and Supplementary Figure S1). Together, these data suggested that LmaH3-GFP is incorporated into the host cell chromatin, although it is not a frequent event.

### Crystal structure of the LmaH3 nucleosome

**Figure 2.**
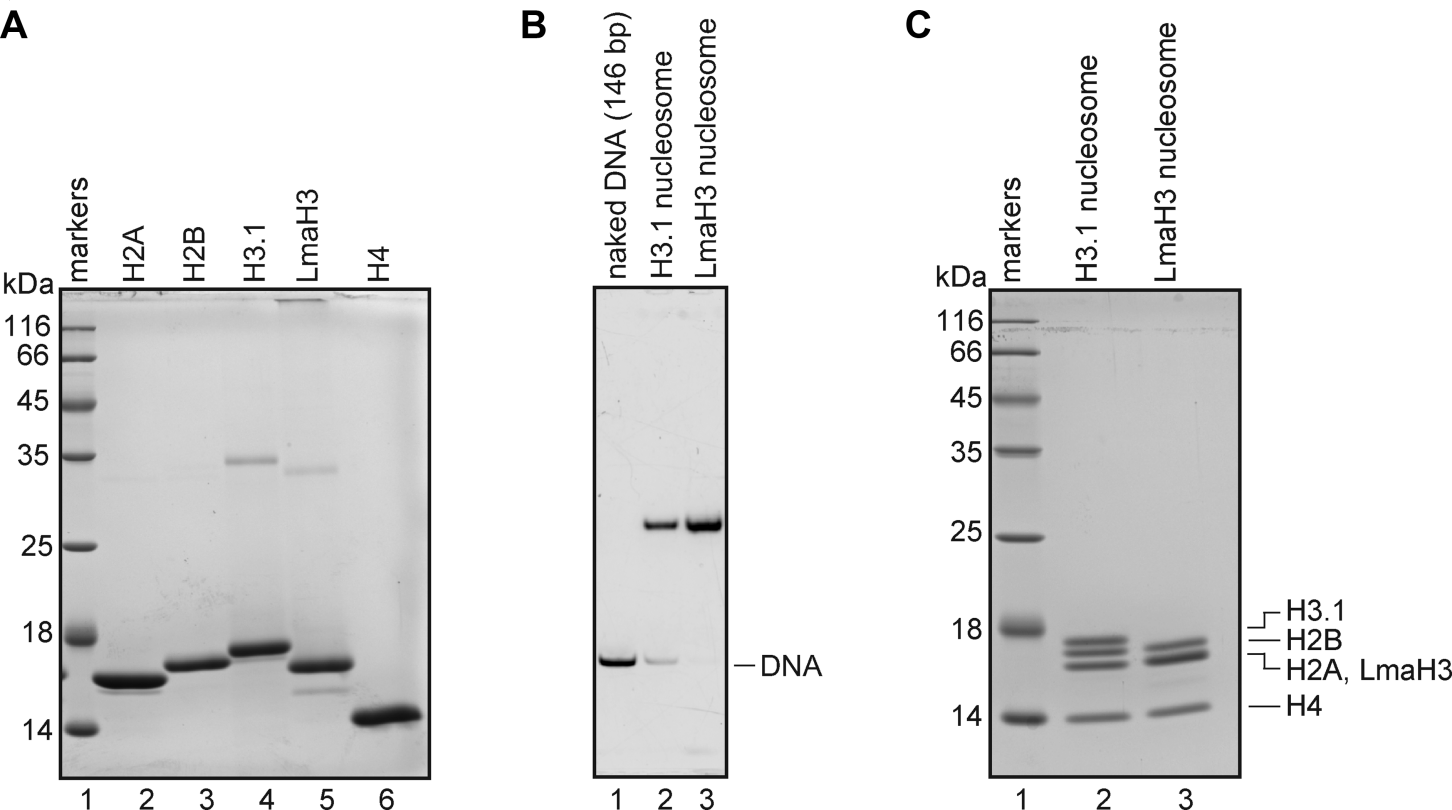
Reconstitution of the LmaH3 nucleosome. (**A**) Preparation of histones. Purified histones H2A, H2B, H3.1, H4 and LmaH3 were analyzed by 18% SDS-PAGE with Coomassie Brilliant Blue (CBB) staining. (**B**) Reconstitution of the H3.1 and LmaH3 nucleosomes. A histone octamer containing H3.1 or LmaH3 (lanes 2 and 3, respectively) was mixed with the palindromic 146 bp satellite DNA fragment (lane 1), and the nucleosomes were reconstituted by the salt dialysis method. The reconstituted nucleosomes were purified using a Prep Cell apparatus, and were analyzed by 0.2× TBE nondenaturing 6% PAGE with ethidium bromide staining. (**C**) The histone contents of the purified H3.1 and LmaH3 nucleosomes were analyzed by 18% SDS-PAGE with CBB staining (lanes 2 and 3, respectively).

**Figure 3.**
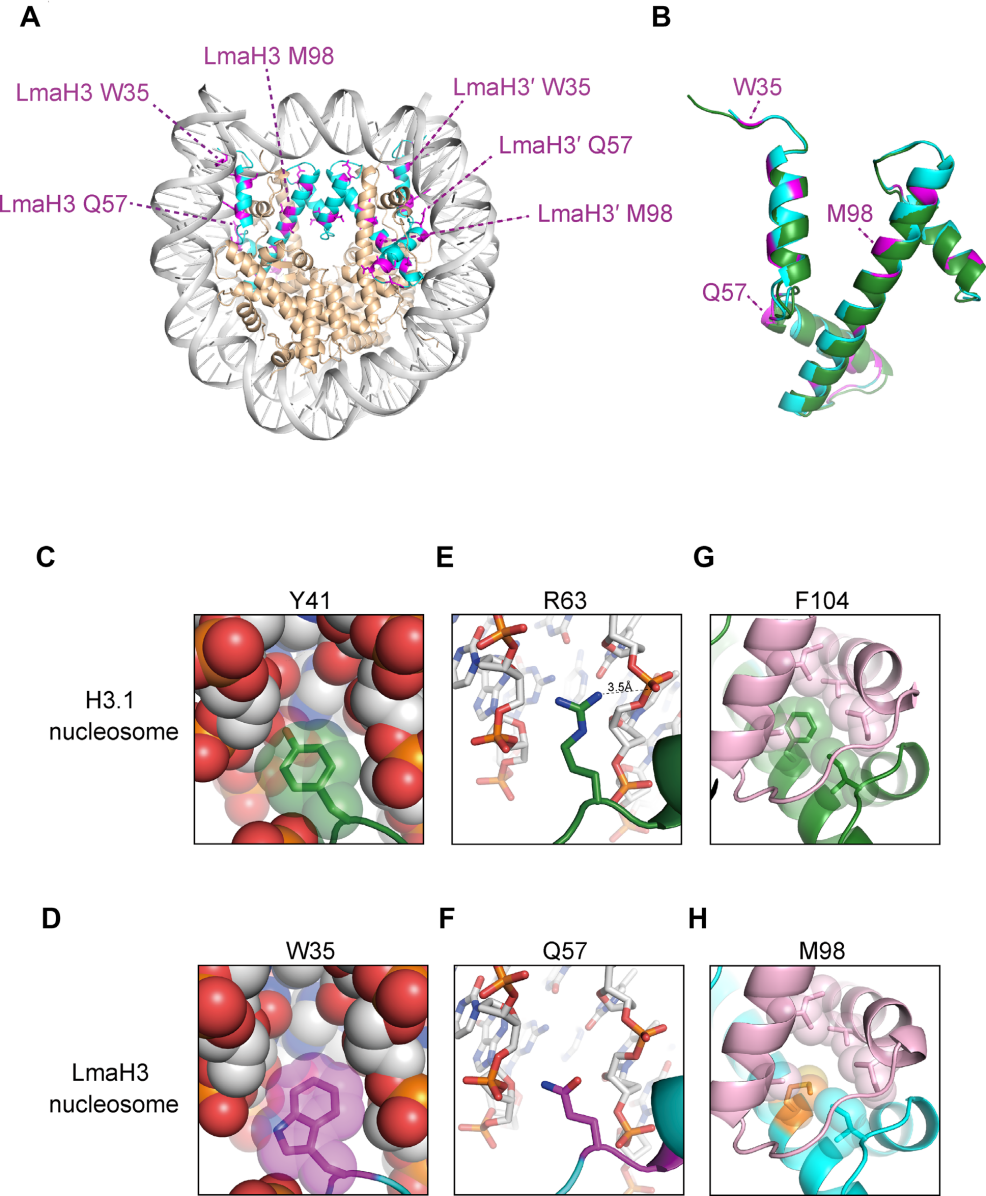
Crystal structure of the LmaH3 nucleosome. (**A**) Overall crystal structure of the LmaH3 nucleosome. The LmaH3 molecules are colored cyan, and the LmaH3-specific residues are shown as magenta with side chains. The LmaH3-specific Trp35, Gln57 and Met98 residues are indicated in magenta letters. The Trp35, Gln57 and Met98 residues from the other LmaH3 molecule are labeled with prime (′). (**B**) Superimposition of the H3.1 (PDB ID: 3AFA) and LmaH3 structures in the nucleosomes. H3.1 and LmaH3 are colored green and cyan, respectively. The LmaH3-specific residues are colored magenta. (**C–H**) Close-up views of the H3.1 Tyr41, Arg63 and Phe104 residues, corresponding to the LmaH3 Trp35, Gln57 and Met98 residues.

We next assessed whether LmaH3 forms a nucleosome with human histones (LmaH3 nucleosome). LmaH3 was purified as a recombinant protein produced in the *E. coli* BL21 (DE3) strain (Figure 2A, lane 5). We performed nucleosome reconstitution by the salt-dialysis method, using purified LmaH3 and human histones H2A, H2B, and H4 in the presence of the palindromic α-satellite 146 bp DNA (26). The LmaH3 nucleosome was successfully reconstituted (Figure 2B). The histone compositions of the LmaH3 and H3.1 nucleosomes were analyzed by SDS-PAGE (Figure 2C). The stoichiometry of the four histones in the H3.1 nucleosome was confirmed (Figure 2C, lane 2). In contrast, three bands were detected for the LmaH3 nucleosome (Figure 2C, lane 3), because the migrations of LmaH3 and human histone H2A were equal (Figure 2A, lanes 2 and 5). We then determined the crystal structure of the LmaH3 nucleosome at 3.63 Å resolution (Supplementary Table S1). In the LmaH3 nucleosome, all of the histones are actually incorporated into the nucleosome (Figure 3A), and the structure of LmaH3 in the nucleosome was similar to that of H3.1 (Figure 3B).

### Histone H3-DNA interactions in the LmaH3 nucleosome

In the crystal structure of the LmaH3 nucleosome, the LmaH3-specific amino acid residues, Trp35 and Gln57, which correspond to the H3.1 Tyr41 and Arg63 residues, are located near the DNA (Figure 3A). In the human H3.1 nucleosome, the side chain of Tyr41 hydrophobically interacts in the minor groove of the DNA (Figure 3C). In the LmaH3 nucleosome, the corresponding residue, Trp35, also interacts in the minor groove, but its hydrophobic interaction surface is different, as compared to the H3.1 Tyr41 surface (Figure 3D, Supplementary Figure S2A). The H3.1 Arg63 residue electrostatically interacts with the DNA backbone via its positively charged side chain moiety (Figure 3E). The side chain of the corresponding LmaH3 Gln57 residue has a neutral charge, suggesting that the interaction with the DNA is weakened in the LmaH3 nucleosome (Figure 3F, Supplementary Figure S2B). The highly conserved H3.1 Phe104 residue, located at the interface of H3.1 and H4, is known to hydrophobically interact with H4 (52) (Figure 3G). The replacement of the Phe104 residue by the corresponding Met98 in the LmaH3 nucleosome may affect the interaction with H4, leading to the reduced stability of the nucleosome (Figure 3H, Supplementary Figure S2C). The LmaH3-specific amino acid residues could influence both the histone–DNA and histone–histone interactions in the nucleosome.

### LmaH3 forms an unstable nucleosome

**Figure 4.**
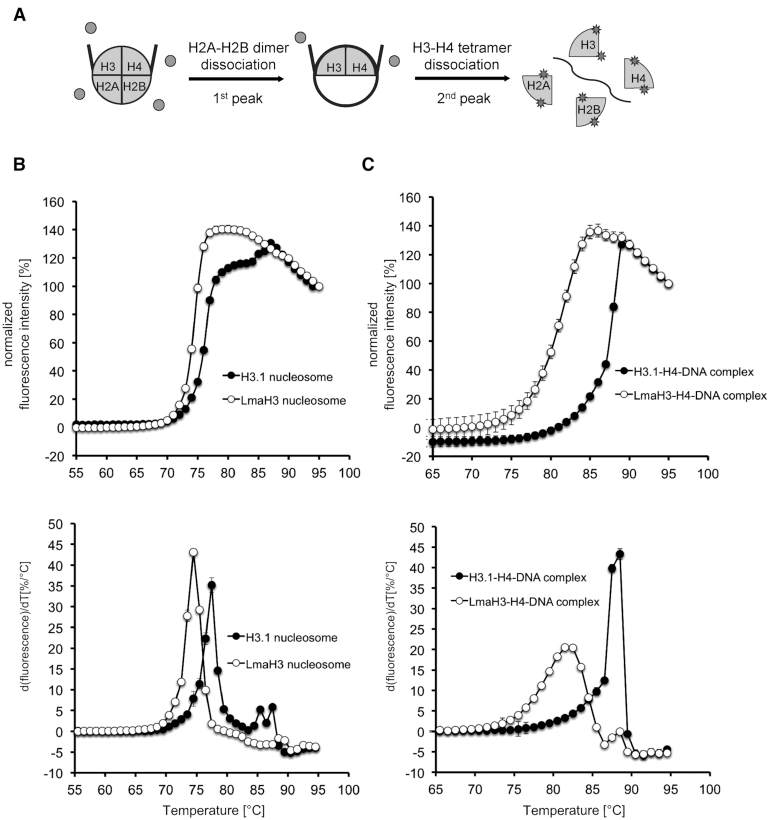
Thermal stability assay of the LmaH3 nucleosome. (**A**) Schematic representation of the nucleosome disruption during the thermal stability assay. In this assay, the fluorescence of SYPRO Orange, a fluorescent dye that hydrophobically binds to denatured histones, is monitored as the histones dissociate from the nucleosomes or the nucleosomes lacking H2A and H2B molecules. (**B**) The upper panel shows the normalized fluorescence intensity curves of the thermal dissociation of the nucleosomes containing H3.1 (•) or LmaH3 (○). The first and second melting temperatures correspond to the dissociations of the H2A–H2B dimers and the H3–H4 tetramer from the nucleosome, respectively. The bottom panel shows the derivative values of the thermal stability curves presented in the upper panel. The bars indicate standard deviations of triplicate experiments. Three independent experiments were performed and similar results were obtained. The H3.1 and LmaH3 nucleosomes were reconstituted with the 145 bp Widom601L DNA. Gel images showing the purified nucleosomes and the nucleosomal histone contents are presented in Supplementary Figure S3A and B. (**C**) The upper panel shows the normalized fluorescence intensity curves of the thermal dissociation of the H3–H4–DNA complexes without the H2A–H2B dimer. The bottom panel shows the derivative values of the thermal stability curves presented in the upper panel. The H3.1–H4–DNA complex (•) and LmaH3–H4–DNA complex (○) are shown. The bars indicate standard deviations of triplicate experiments. The H3.1–H4–DNA and LmaH3–H4–DNA complexes without the H2A–H2B dimer were reconstituted with the 145 bp Widom601L DNA. Gel images showing the purified histone–DNA complexes and the histone contents are presented in Supplementary Figure S3C and D.

We conducted thermal stability assays to test the stability of the LmaH3 nucleosome (Figure 4A, and Supplementary Figure S3A and B). In the control H3.1 nucleosome (Figure 4B), the histone dissociation is characterized by two melting transition temperatures (Tms), which correspond to the H2A–H2B and H3–H4 dissociations, respectively. In contrast, in the LmaH3 nucleosome, the two Tms could not be separately observed, and the stability of the LmaH3 nuclosome was clearly decreased (Figure 4B). In order to eliminate the signal from H2A–H2B dissociation, we reconstituted the H3–H4–DNA and LmaH3–H4–DNA complexes (Supplementary Figure S3C and D) and performed the thermal stability assay. We found that LmaH3–H4 dissociated from the DNA at a remarkably lower temperature than H3.1–H4 (Figure 4C). Therefore, we concluded that the LmaH3 is incorporated into the nucleosome, although the LmaH3–H4 association with DNA is weaker, as compared to the human H3.1–H4–DNA complex.

### LmaH3-specific amino acid residues affect LmaH3 nucleosome stability

**Figure 5.**
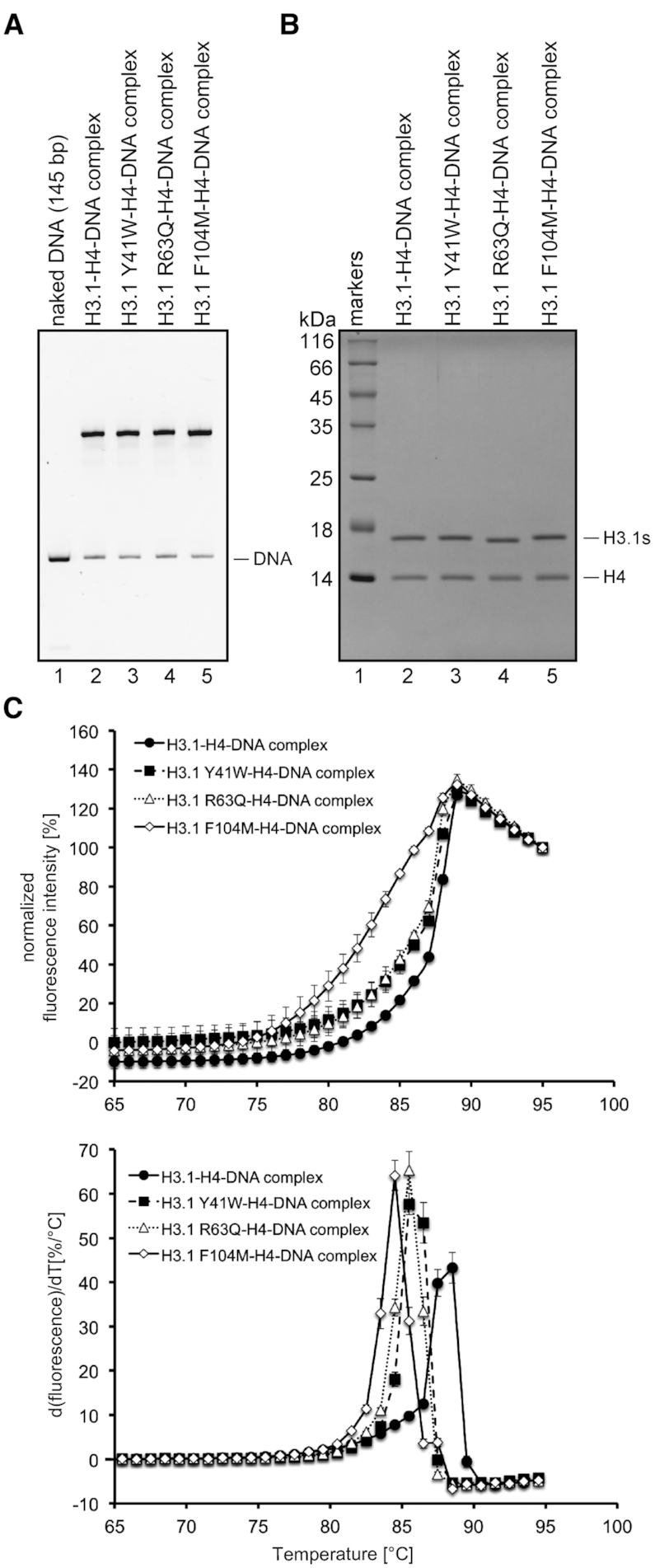
Thermal stability assay of the H3.1 Y41W–H4–DNA, H3.1 R63Q–H4–DNA and H3.1 F104M–H4–DNA complexes. (**A**) Reconstitution of the H3.1–H4–DNA, H3.1 Y41W–H4–DNA, H3.1 R63Q–H4–DNA and H3.1 F104M–H4–DNA complexes. H3.1, H3.1 Y41W, H3.1 R63Q and H3.1 F104M (lanes 2, 3, 4, and 5, respectively) were each mixed with the palindromic 145 bp satellite DNA fragment (lane 1), and the nucleosomes were reconstituted by the salt dialysis method. The reconstituted complexes were purified using a Prep Cell apparatus, and were analysed by 0.2× TBE nondenaturing 6% PAGE with ethidium bromide staining. (**B**) The histone contents of the purified H3.1–H4–DNA, H3.1 Y41W–H4–DNA, H3.1 R63Q–H4–DNA and H3.1 F104M–H4–DNA complexes were analysed by 16% SDS-PAGE with CBB staining (lanes 2, 3, 4, and 5 respectively). (**C**) The upper panel shows the normalised fluorescence intensity curves of the thermal dissociation of the H3.1–H4–DNA (•), H3.1 Y41W–H4–DNA (▓), H3.1 R63Q–H4–DNA (△) and H3.1 F104M–H4–DNA (◇) complexes. The bottom panel shows the derivative values of the thermal stability curves presented in the upper panel. The bars indicate standard deviations of triplicate experiments.

We then performed mutation analyses to test whether the H3.1 Tyr41, Arg63 and Phe104 residues, described in Figure 3, contribute to the instability of the LmaH3 nucleosome. The H3.1 Y41W, H3.1 R63Q and H3.1 F104M mutants were prepared, in which the H3.1 Tyr41, Arg63 and Phe104 residues were replaced by the corresponding LmaH3 Trp35, Gln57 and Met98 residues, respectively. The H3.1–H4–DNA complexes containing the H3.1 Y41W, H3.1 R63Q and H3.1 F104M mutants were then successfully reconstituted by salt dialysis (Figure 5A and B). We performed a thermal stability assay to evaluate the stability of the H3.1 Y41W–H4–DNA, H3.1 R63Q–H4–DNA and H3.1 F104M–H4–DNA complexes. The disruption of the H3.1 Y41W–H4–DNA and H3.1 R63Q–H4–DNA complexes occurred at a moderately lower temperature, as compared to the control H3.1–H4–DNA complex (Figure 5C). On the other hand, the H3.1 F104M–H4–DNA complex dissociation occurred at a remarkably lower temperature, as compared to the other two mutants (Figure 5C). These results indicated that the replacement of the H3.1 Tyr41, H3.1 Arg63 and H3.1 Phe104 residues with the corresponding LmaH3 residues weakened the H3.1–H4 association with DNA. Therefore, the specific LmaH3 Trp35, Gln57 and Met98 residues may be responsible for the instability of the LmaH3–H4–DNA complex, although the contributions of the LmaH3 Trp35 and Gln57 residues and the LmaH3 Met98 residue to the unstable nature of the LmaH3 nucleosome may be different (see discussion).

### The LmaH3 nucleosome maintains a relaxed chromatin conformation

**Figure 6.**
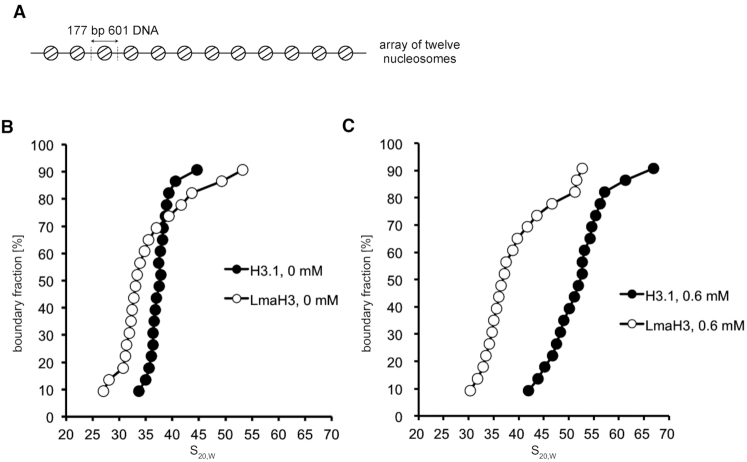
The LmaH3 nucleosome maintains an open chromatin conformation. (**A**) Schematic representation of the nucleosome array reconstituted with 12 repeats of the 177 bp Widom601 DNA. (B and C) Sedimentation velocity analyses of the nucleosome arrays containing the H3.1 (•) or LmaH3 (○) nucleosome, in the absence (**B**) or presence (**C**) of 0.6 mM MgCl_2_. The enhanced van Holde–Weischet method was used to determine the distribution of the sedimentation coefficients. Three independent experiments were performed and similar outcomes were obtained, using nucleosomal arrays prepared by two independent reconstitutions, shown in Supplementary Figure S4E and F.

The biochemical properties of the mononucleosome have been shown to influence the higher order chromatin conformation *in vitro* (45,50,53). Therefore, we tested the influence of the LmaH3 nucleosome on higher order chromatin folding by analytical sedimentation velocity ultracentrifugation (50). Arrays consisting of 12 nucleosomes were assembled with histone octamers containing H3.1 or LmaH3 on tandem repeats of the Widom601 DNA sequence (177 bp) (Figure 6A, Supplementary Figure S4A and B). The nucleosome occupancies of the reconstituted nucleosome arrays were estimated by digestion with the restriction enzyme *Sca*I, followed by native polyacrylamide gel electrophoresis; the reconstituted arrays yielded undetectable amounts of the free 601 DNA (Supplementary Figure S4C and D). Sedimentation studies of the nucleosome arrays were then performed in the absence of Mg^2+^ ion (Figure 6B). The control H3.1 nucleosome array showed sedimentation values consistent with recently published data, obtained with a sample prepared by the same method (Figure 6B) (54). Interestingly, we observed lower sedimentation values for the LmaH3 nucleosome array, as compared to the H3.1 nucleosome array (Figure 6B). These results indicated that the LmaH3 nucleosome array adopts a more relaxed chromatin conformation than the H3.1 nucleosome array. The addition of Mg^2+^ ion reportedly causes the nucleosome array to adopt a higher folded state (55,56). Therefore, we performed the sedimentation assay in the presence of 0.6 mM MgCl_2_ (Figure 6C). In contrast to the H3.1 nucleosome array, in the presence of 0.6 mM MgCl_2_, the sedimentation profile of the LmaH3 nucleosome array was similar to the profile observed in the absence of Mg^2+^ ions (Figure 6B and C). These results indicated that the LmaH3 nucleosome array preserves the relaxed chromatin conformation, even in the presence of Mg^2+^ ion.

## DISCUSSION

Histones are fundamental chromatin proteins that play an important role in DNA packaging and regulation of gene expression in eukaryotes. Protozoan parasite infections promote epigenetic changes to the host cell chromatin (6,7). Indeed, transcriptional changes in infected host cells have been reported in numerous studies (6,57). The parasitic infection may affect the chromatin structure, but its mechanism has remained enigmatic.

Histones have been identified among the proteins secreted from cells infected by not only *Leishmania* (17–20), but also other parasites such as *Toxoplasma*, *Plasmodium*, and the fungal eukaryotic pathogens *Cryptococcus* and *Histoplasma* (21–24,58,59). Therefore, we anticipated that parasite histones could play a role in modulating the host chromatin. In the present study, we found that the *Leishmania* histone H3, LmaH3, is incorporated into human chromatin *in vivo* (Figure 1), and formed nucleosomes with human histones *in vitro* (Figures 2 and 3), although *Leishmania* histone incorporation into infected cells has not been investigated. The LmaH3 nucleosome displays distinct biochemical properties, such as decreased stability and resistance to chromatin compaction by Mg^2+^ ion (Figures 4–6). In the thermal stability assay, the Tm of the first peak for the LmaH3 nucleosome was 3°C lower than that of the H3.1 nucleosome (Figure 4B). However, the Tm of the LmaH3–H4–DNA complex was 7°C lower, as compared to that of the H3.1–H4–DNA complex (Figure 4C). This indicated that the presence of H2A–H2B enhanced the stability of LmaH3 incorporation into the human nucleosome. We also found that the mutations of the H3.1 Tyr41 and Arg63 residues, corresponding to the LmaH3 Trp35 and Gln57 residues, respectively, moderately decreased the stability of the H3.1–H4–DNA complex. On the other hand, the mutation of the H3.1 Phe104 residue, corresponding to the LmaH3 Met98 residue, drastically decreased the stability of the H3.1–H4–DNA complex (Figure 5C). The crystal structure suggested that the LmaH3-specific Trp35 and Gln57 residues weaken the interaction with DNA, while the LmaH3-specific Met98 residue affects the H3–H4 interaction (Figure 3 and Supplementary Figure S2). These facts implied that the H3–H4 association might play an important role for the stable incorporation of LmaH3 into the nucleosome.

These characteristics of the LmaH3 nucleosome may modulate the host gene expression, allowing the persistence of the parasite in host cells. Therefore, these observations support the possibility that histones are secreted intentionally by parasites during infection, to modulate the host chromatin structure. Marr *et al.* (60) reported that *Leishmania* infection triggered changes in the host cell DNA methylation patterns on genes involved in macrophage defenses, to facilitate the establishment of the parasite and its survival (60). DNA methylation is generally associated with gene suppression (61,62). *Leishmania* histones may alter the host DNA epigenetic status through changing the higher order chromatin structure, and may suppress unfavorable host genes to facilitate parasite survival.

Based on our results and previous findings, we propose a new parasite virulence mechanism involving LmaH3 during host cell infection. In this mechanism, the secreted LmaH3 forms a nucleosome with the human histones in the host cell chromatin during *Leishmania* parasite infection. The resulting hybrid nucleosome with parasite and human histones maintains a relaxed conformation of the chromatin. This relaxed chromatin folding may alter the epigenetic state and gene expression pattern to optimize *Leishmania* parasite establishment and survival. The incorporation efficiency of LmaH3 in the host chromatin is low; however, its impact on the nucleosome stability and chromatin compaction is robust. This suggests that the secreted LmaH3 may have a substantial effect on the higher order chromatin configuration in the infected cells. The *Leishmania* Nap protein is reportedly a secreted protein (17). This histone chaperone might play a role in the LmaH3 exchange mechanism, and may increase the LmaH3 incorporation efficiency *in vivo*. Therefore, the nucleosome containing LmaH3 may become a possible drug target for anti-leishmanial chemotherapy. Further studies are awaited.

## DATA AVAILABILITY

The atomic coordinates of the LmaH3 nucleosome have been deposited in the Protein Data Bank, with the PDB ID 6KXV.

## Supplementary Material

gkz1040_Supplemental_File

## Notes

Present address: Naoki Horikoshi, Life Science Center for Survival Dynamics, University of Tsukuba, Tsukuba, Ibaraki, Japan.
