# Supplementary material for: Incorporation and influence of Leishmania histone H3 in chromatin
Source: Nucleic Acids Res. 2019 Nov 13;47(22):11637–48. doi: 10.1093/nar/gkz1040 (PMC7145708; doi:10.1093/nar/gkz1040)
Supplement: gkz1040_Supplemental_File [file gkz1040_supplemental_file.pdf]

Supplementary Information

Incorporation and influence of *Leishmania* histone H3 in chromatin

Mariko Dacher[1], Hiroaki Tachiwana[2], Naoki Horikoshi[3+], Tomoya Kujirai[1], Hiroyuki Taguchi[3], Hiroshi Kimura[4] and Hitoshi Kurumizaka[1,3]*

[1]Laboratory of Chromatin Structure and Function, Institute for Quantitative Biosciences, The University of Tokyo, 1-1-1 Yayoi, Bunkyo-ku, Tokyo 113-0032, Japan.

[2]Department of Cancer Biology, The Cancer Institute of Japanese Foundation of Cancer Research, 3-8-31 Ariake, Koto-ku, Tokyo 135-8550, Japan.

[3]Graduate School of Advanced Science and Engineering, Waseda University, 2-2 Wakamatsu-cho, Shinjuku-ku, Tokyo 162-8480, Japan.

[4]Cell Biology Center, Institute of Innovative Research, Tokyo Institute of Technology, 4259 Nagatsuta-cho, Midori-ku, Yokohama 226-8503, Japan.

*To whom correspondence should be addressed. Tel: +81-3-5841-7826;
Fax: +81-3-5841-1468; Email: kurumizaka@iam.u-tokyo.ac.jp

[+] Present address: Naoki Horikoshi, Life Science Center for Survival Dynamics, University of Tsukuba, Tsukuba, Ibaraki, Japan

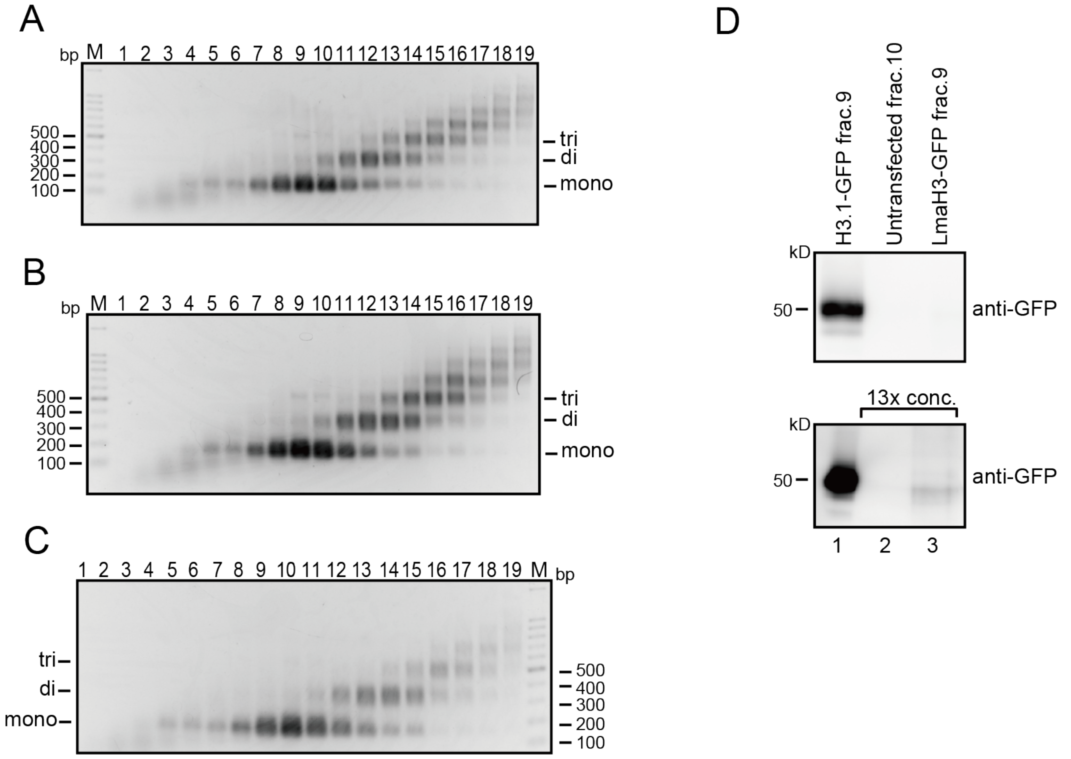

Supplementary Figure S1. Sucrose gradient ultracentrifugation. The chromatin fractions from HeLa cells stably expressing LmaH3-GFP (**A**), H3.1-GFP (**B**), or untransfected cells (**C**) were subjected to sucrose gradient ultracentrifugation. The resulting DNA fragments were analyzed by agarose gel electrophoresis with ethidium bromide staining. The DNA markers are indicated as M. (**D**) Detection of LmaH3-GFP by a western blotting analysis. The presence of H3.1-GFP and LmaH3-GFP was detected by a western blotting analysis, using the anti-GFP monoclonal antibody. Samples of HeLa cells expressing H3.1-GFP (fraction 9), untransfected cells (fraction 10), and HeLa cells expressing LmaH3-GFP (fraction 10) were concentrated, and equivalent sample quantities were subjected to the western blotting (upper panel). To detect the low amount of LmaH3 incorporated into chromatin, the samples (fraction 9) from LmaH3-GFP cells and untransfected cells (as a negative control) were concentrated 13-fold, and then subjected to western blotting. The quantity of the sample expressing H3.1-GFP (fraction 9) was the same as that used in the upper panel. The molecular weights of the marker proteins are indicated. The full gel images of Supplementary Figure S1A-D are presented in Supplementary Figures S5 F-J, respectively.

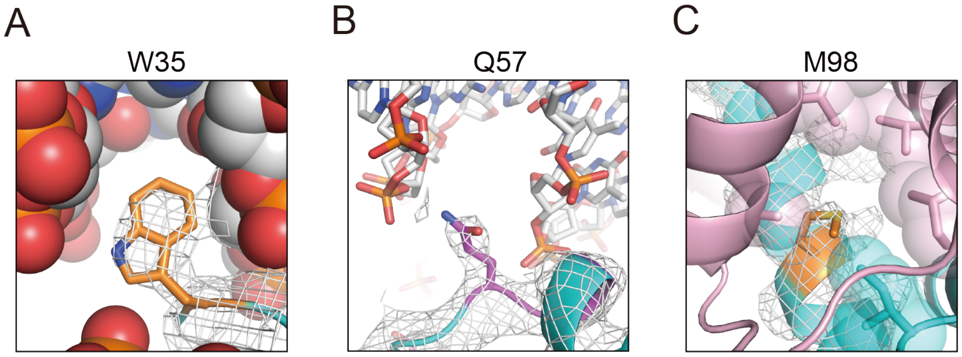

Supplementary Figure S2. The 2mFo-DFc electron density maps of the LmaH3 Trp35 (A), Gln57 (B), and Met98 (C) residues. The electron density maps are presented at 1 σ.

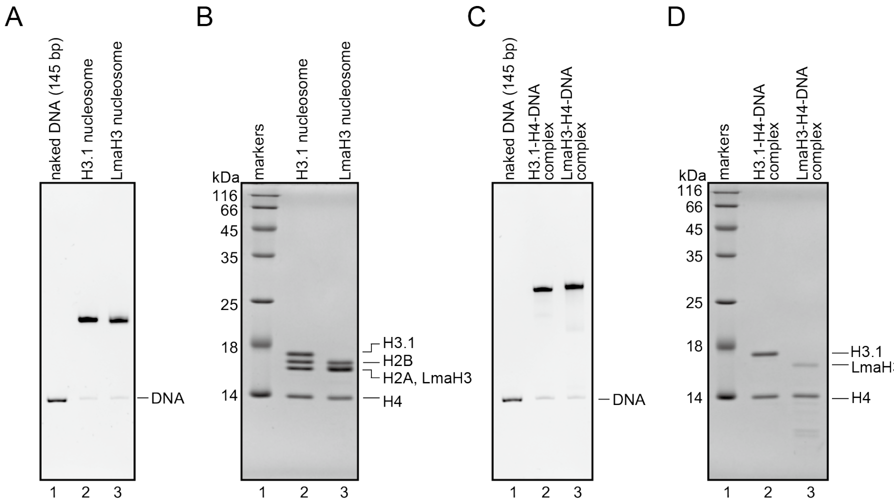

Supplementary Figure S3. (A) A histone octamer containing H3.1 or LmaH3 was mixed with the 145 bp Widom601L DNA, and the nucleosomes were reconstituted by the salt dialysis method. The reconstituted nucleosomes were purified using a Prep Cell apparatus, and were analyzed by 0.2x TBE nondenaturing 6% PAGE with ethidium bromide staining. (**B**) The histone contents of the purified H3.1 and LmaH3 nucleosomes were analyzed by 20% SDS-PAGE with CBB staining (lanes 2 and 3, respectively). (**C**) The H3.1-H4-DNA or LmaH3-H4-DNA complexes, without the H2A-H2B dimers, were reconstituted in the presence of the 145 bp Widom601L DNA. The resulting complexes were purified and analyzed by 0.2x TBE nondenaturing 6% PAGE with ethidium bromide staining. (**D**) The histone contents of the purified complexes with H3.1-H4 or LmaH3-H4 were analyzed by 20% SDS-PAGE with Coomassie Brilliant Blue staining.

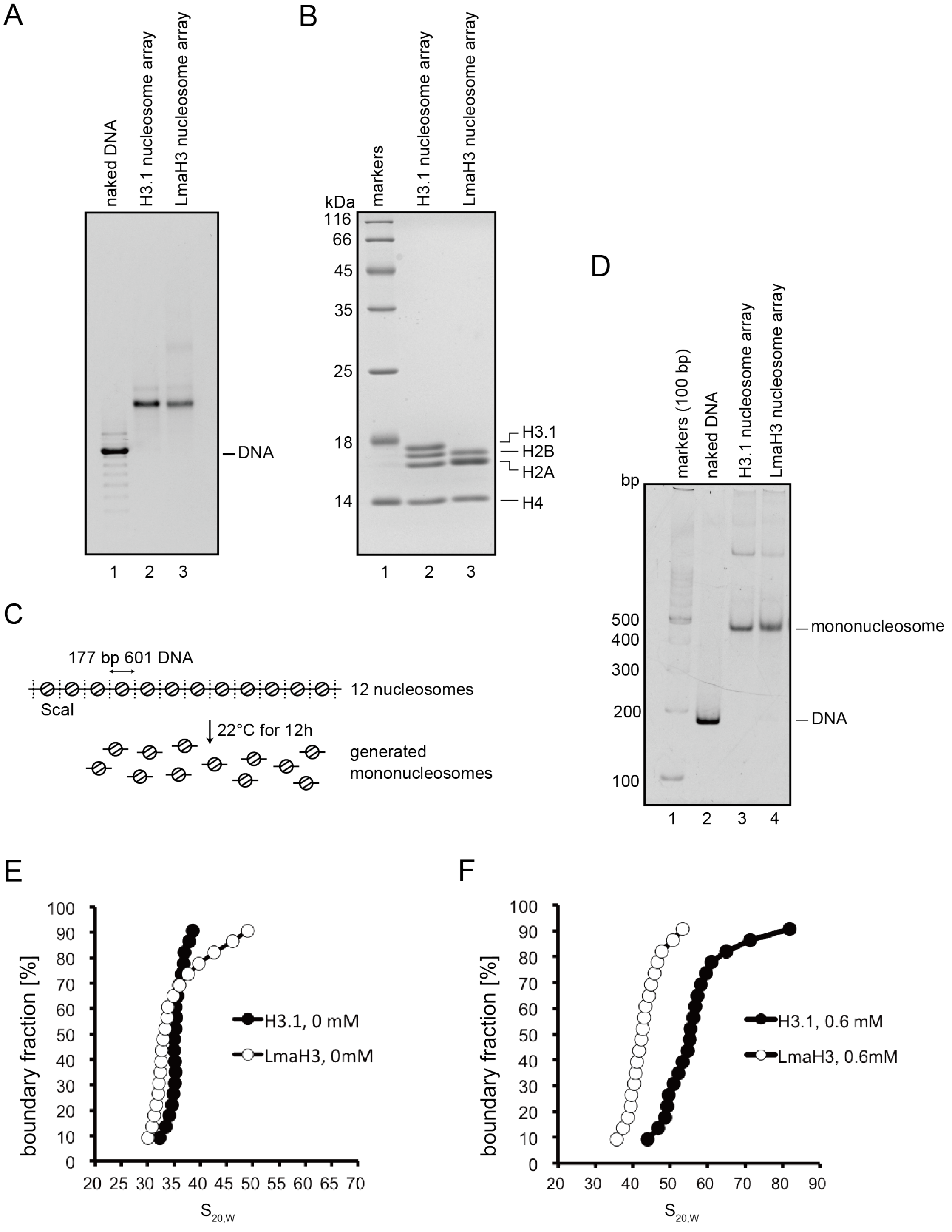
A
naked DNA
H3.1 nucleosome array
LmaH3 nucleosome array
DNA
1 2 3
B
markers
H3.1 nucleosome array
LmaH3 nucleosome array
kDa
116
66
45
35
25
18
14
H3.1
H2B
H2A
H4
1 2 3
C
177 bp 601 DNA
ScaI
12 nucleosomes
22°C for 12h
generated
mononucleosomes
D
markers (100 bp)
naked DNA
H3.1 nucleosome array
LmaH3 nucleosome array
bp
500
400
300
200
100
mononucleosome
DNA
1 2 3 4
E
boundary fraction [%]
H3.1, 0 mM
LmaH3, 0mM
S20,W
F
boundary fraction [%]
H3.1, 0.6 mM
LmaH3, 0.6mM
S20,W

Supplementary Figure S4. Reconstitution of nucleosome arrays containing H3.1 or LmaH3. (**A**) The purified 12 repeats of the 177 bp Widom601 DNA and the reconstituted H3.1 and LmaH3 nucleosome arrays were analyzed by 0.7% agarose gel electrophoresis with ethidium bromide staining. (**B**) The histone compositions of the purified H3.1 (lane 2) and LmaH3 (lane 3) nucleosome arrays were analyzed by 18% SDS-PAGE with Coomassie Brilliant Blue staining. (**C**) Schematic representation of the *Sca*I digestion of the nucleosome array. The nucleosome array reconstituted with 12 repeats of the 177 bp Widom601 DNA was digested at 22°C for 12 h by *Sca*I, which cleaves the linker regions between nucleosomes, thus generating mononucleosomes. (**D**) Nucleosome occupancy of the reconstituted nucleosome arrays. The template DNA and the reconstituted nucleosome arrays containing H3.1 or LmaH3 were completely digested by *Sca*I, and the resulting mononucleosomes were analyzed by nondenaturing 5% PAGE with ethidium bromide staining (lanes 2-4). (**E and F**) Analytical ultracentrifugal sedimentation velocity analyses of the H3.1 (●) and LmaH3 (○) nucleosome arrays, prepared from independent reconstitutions in the absence (**E**) or presence (**F**) of 0.6 mM $MgCl_2$. The enhanced van Holde-Weischet method was used to determine the distribution of the sedimentation coefficients.

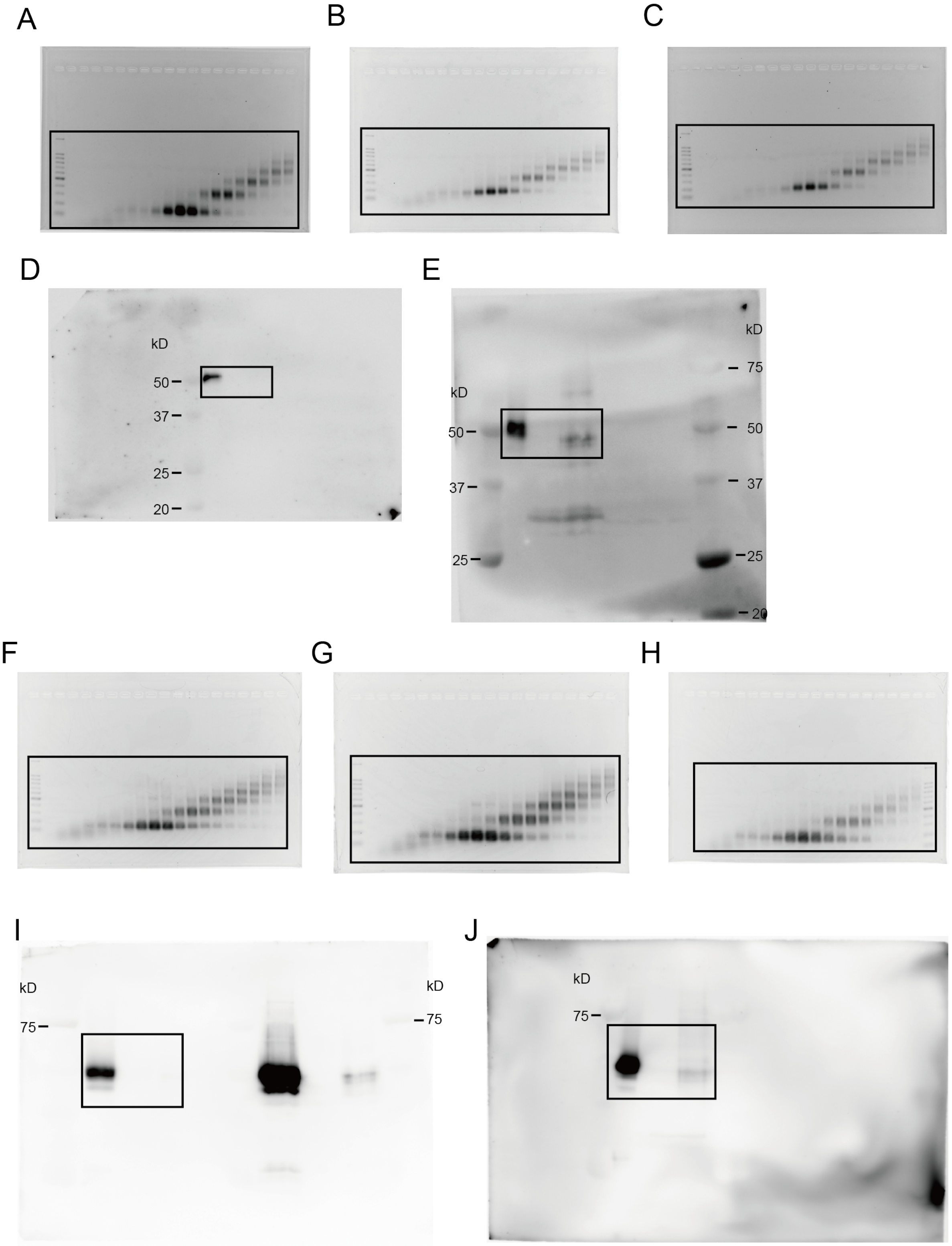
A
B
C
D
kD
50
37
25
20
E
kD
75
50
37
25
20
kD
50
37
25
F
G
H
I
kD
75
kD
75
J
kD
75

Supplementary Figure S5. Full images. (**A**) The full image of Fig. 1C. (**B**) The full image of Fig. 1D. (**C**) The full image of Fig. 1E. (**D**) The full image of Fig. 1F upper panel. (**E**) The full image of Fig. 1F lower panel. (**F**) The full image of Fig. S1A. (**G**) The full image of Fig. S1B. (**H**) The full image of Fig. S1C. (**I**) The full image of Fig. S1D upper panel. (**J**) The full image of Fig. S1D lower panel. Black rectangles indicate the areas presented in the figures. The molecular weights of the marker proteins are indicated

Supplementary Table S1. Data collection and refinement statistics

	LmaH3 nucleosome
**Data Collection**	
Space Group	$P2_12_12_1$
Wavelength	1.100000
Cell Dimensions	
a (Å)	100.868
b (Å)	101.300
c (Å)	174.981
α, β, γ (°)	90.000 90.000 90.000
Resolution (Å)	48.9-3.63 (3.85-3.63)
Reflections (Unique)	21054
R_{merge} (%)	23.2 (222.6)
R_{meas} (%)	23.8 (228.4)
$I/\sigma(I)$	10.57 (1.34)
Completeness (%)	99.6 (97.7)
$CC_{1/2}$ in outer shell	0.515
Redundancy	20.99 (20.00)
**Refinement**	
Resolution (Å)	48.9 - 3.63
R_{work}/R_{free} (%)	22.0 / 26.4
No. atoms	11876
Protein	5896
DNA	5980
B factors	
Protein	101.2
DNA	154.0
R.m.s. deviations	
Bond lengths (Å)	0.003
Bond angles (°)	0.583
Ramachandran plot	
Favored (%)	97.4
Allowed (%)	2.6
Outliers (%)	0.0
PDB ID	6KXV

Values in parentheses are for the highest resolution shell (3.85-3.63)
